# Midwives' experiences with providing home‐based postpartum care during the COVID‐19 pandemic: A qualitative study

**DOI:** 10.1002/nop2.1986

**Published:** 2023-08-31

**Authors:** Hanne Marie Akselsen, Emilie Hanssen Leknes, Tone Engen, Eline Skirnisdottir Vik, Anne Britt Vika Nilsen

**Affiliations:** ^1^ Department of Health and Caring Sciences Western Norway University of Applied Sciences Bergen Norway

**Keywords:** home visits, maternity nursing, midwifery, post‐partum, qualitative approaches

## Abstract

**Aim:**

To explore midwives' experiences with providing home‐based postpartum care during the COVID‐19 pandemic in Norway.

**Design:**

A descriptive and explorative qualitative study.

**Methods:**

The study is based on semi‐structured individual interviews with 11 midwives experienced in offering home‐based postpartum care. We explored their experiences of such care during the first wave of the COVID‐19 pandemic. Data collection occurred from October through November 2020. An inductive thematic analysis was performed using Systematic Text Condensation (STC) by Malterud (2012).

**Results:**

The following two main results emerged from the analyses: (1) the midwives adapted quickly to changes in postpartum care during the pandemic and (2) midwives saw the experience as an opportunity to re‐evaluate their practices.

**Conclusion:**

This study highlights midwives' resilience and adaptability during the first wave of COVID‐19 pandemic. It emphasises the crucial role of face‐to‐face interactions in postpartum care, while recognising the value of technology when direct access is limited. By shedding light on midwives' experiences, this research contributes to improving postpartum care in unforeseen circumstances. It underscores the significance of interdisciplinary integration in planning postpartum care services and the lasting influence of lessons learned on addressing future challenges.

**Implications for Practice:**

The valuable insights gained from lessons learned during the COVID‐19 pandemic may have a lasting influence on the postpartum care system, empowering it to tackle unforeseen challenges both today and in the future.

**Impact:**

The current study addressed midwives' experience with providing home‐based postpartum care during the COVID‐19 pandemic in Norway. Midwives received an opportunity to re‐evaluate their own practices and valued being included when changes were implemented. The current findings should alert policy makers, leaders and clinicians in postpartum care services when planning future practice.

## 1 INTRODUCTION

Pregnancy, childbirth and the postpartum period represent life events that play a major role in many people's lives (Worlds Health Organization (WHO), 2022). The duties required of the midwives' postpartum home visits include providing breastfeeding guidance, observing the newborn baby, capturing symptoms of postpartum depression and supporting the woman and her family (Eberhard‐Gran et al., 2022; WHO, 2022). This is a particularly vulnerable time for new families, and studies have shown that the COVID‐19 pandemic reinforced this vulnerability for new parents and their babies (Bradfield, Hauck, et al., 2021; Chmielewska et al., 2021; Eberhard‐Gran et al., 2022; Eri et al., 2022). As a result, there is a need for more knowledge regarding the rapid changes we have seen in maternity care services during the COVID‐19 pandemic (van den Berg et al., 2022).

Early discharge policies after childbirth have become increasingly common worldwide (Jones et al., 2021). The prevailing recommendations based on research prior to the COVID‐19 pandemic were that healthy mothers and babies were discharged 4–24 h after birth, depending on the health and well‐being of both mother and baby, the level of support available after discharge and the mothers' preferences (National Institute for Health and Care Excellence (NICE), 2021).

When the COVID‐19 pandemic hit in March 2020, the Norwegian government imposed infection control measures as advised by the Norwegian Directorate of Health and the Norwegian Institute of Public Health. Social distancing, travelling restrictions and lockdown of non‐vital services, both private and public were implemented (Norwegian Directorate of Health, 2020). Restrictions on hospital admittance, presence of partners and length of stay postpartum led to a rise in the level of early discharge in many countries, including Norway (Buek et al., 2022; Eberhard‐Gran et al., 2022; Townsend et al., 2021). A consequence of early discharge policies was that of the increasing demand for home‐based postpartum care services, which further influenced the day‐to‐day work and responsibilities of those who provided these services.

The COVID‐19 regulations provoked unfortunate ripple effects, including restrictions on healthcare services such as home‐based postpartum care. Examples of how the pandemic may have had a detrimental influence on this kind of care at the time include a negative impact on breastfeeding, as can be seen in the fact that more than 30% of women who gave birth in Europe during the pandemic reported inadequate breastfeeding support (Eberhard‐Gran et al., 2022; Eri et al., 2022; Lazzerini et al., 2022; Silverio et al., 2021). On the other hand, early discharge during the pandemic may have been beneficial to some families, as the non‐birthing parent and other family members at home were given the opportunity to participate more when the mother and baby received their care in the family home (Johansson et al., 2022). The COVID‐19‐related regulations did, however, also impact the professional practices of the midwives, as both the structure and the process of the home‐based postpartum care were profoundly affected by these new rules (Eberhard‐Gran et al., 2022; Renfrew et al., 2020; Townsend et al., 2021).

## 2 BACKGROUND

The challenge of quickly adjusting and adhering to new and rapidly changing guidelines on infection control, while still providing high‐quality care, has in a series of recent studies been described as stressful by health workers (Ardebili et al., 2021; Bradfield, Wynter, et al., 2021; Fumagalli et al., 2022; González‐Timoneda et al., 2021). Yet even though there is a growing number of studies reporting on the experiences of new families in regard to the home‐based postpartum care they received during the pandemic, less is known about the experiences of the midwives who actually provided home‐based postpartum care.

## 3 THE STUDY

### 3.1 Aim

The aim of the study was to explore midwives' experiences with providing home‐based postpartum care during the COVID‐19 pandemic in Norway.

## 4 METHODS

### 4.1 Design

The study is of a qualitative design, analysed by an inductive thematic analysis using Systematic Text Condensation (STC) (Malterud, 2012).

### 4.2 Sampling and recruitment

The midwives were recruited via the closed Facebook group ‘Midwives in Norway’. Midwives who provided home‐based postpartum care in Norway before and during the COVID‐19 pandemic were eligible for inclusion. To be included in the study, potential participants contacted the researchers by phone or e‐mail.

Initially, we intended to include all midwives who met the inclusion criteria and asked to participate in the study on a rolling basis (convenience sampling). However, as many midwives showed interest, we took a more a strategic approach (purposive sampling), actively choosing midwives from different municipalities and regions of Norway to achieve variation within the experiences to be explored (Malterud et al., 2016).

### 4.3 Data collection

A semi‐structured interview guide (see Supporting Information) was developed and used during the interviews, and a pilot interview was conducted before the data collection took place. The interviews were conducted from October through November 2020.

We carried (HMA, EHL) out a total of 11 individual semi‐structured interviews. Ten of these were conducted via a secure internet connection (i.e., Zoom Cloud Meetings hosted by Uninett for the Norwegian educational sector), and one was conducted at a health centre, by request of the participant. The interviews lasted between 22 and 60 min (average: 40 min). The interviews were recorded on tape and the raw data was stored in an assigned research server at the University. The equipment used for the audio recordings was approved and owned by the library services at the University. All the interviews were conducted and transcribed in Norwegian by HMA and EHL and later translated to English by the authors of the current paper. The information power was assessed as sufficient; therefore, no additional interviews were conducted (Malterud et al., 2016).

### 4.4 Data analysis

Four researchers (HMA, EHL, ESV, ABVN) analysed the data using STC: a four‐step cross‐case analysis method (Malterud, 2012). In the first step of the analysis, we listened and read through the interviews repeatedly in order to immerse ourselves in the data material. In this way, we gained an overall impression of the data and found preliminary themes relevant to the aim of the study. In the second step of the analysis, we decontextualised the data material by identifying meaning units related to midwives' experiences with providing postpartum care during the first wave of the COVID‐19 pandemic. These meaning units were later coded and organised into code groups. In the third step, we dealt with one group of code at a time and further organised the content of each code group into subgroups. The meaning of the subgroups was abstracted using text condensation. In the fourth step, we summarised the condensates of the analytical text. Finally, we identified quotations to illustrate the summarised text (i.e., a golden quotation). The analysis process was data driven, and Donabedian's framework for quality of care (Donabedian, 2005) was used in discussing the results.

### 4.5 Ethical considerations

This study was approved by the Regional Committees for Medical and Health Research Ethics (REK) (ref. no. 25044) and the Norwegian Centre for Research Data AS (NSD) (ref. no. 544007). Before the interviews, the midwives who were recruited to the study were sent an information letter. Those who wished to participate gave written informed consent before the interviews were conducted. The participants were informed that they had the opportunity to withdraw from the study, without this having negative consequences for them.

### 4.6 Trustworthiness

The quality of the study was evaluated using the concepts of validity, relevance and reflexivity (Malterud, 2017). The purpose, design and data collection methods of the study were relevant to the research objectives and suitable for providing valid answers to the questions we asked. By continuously questioning the validity of the study and following the principle of reflexivity by being aware of our own preconceptions, we aimed to enhance the trustworthiness of the study (Malterud, 2017).

In the current study, the first and second authors took field notes and kept a research diary throughout the process involved in collecting and analysing the data. All interviews were conducted by the first and second authors as a part of their master's degree in midwifery. In line with STC (Malterud, 2012), four of the authors (HMA, EHL, ESV, ABVN) repeatedly went back and forth between the four steps of the analysis. According to Malterud (2012), step‐by‐step analysis of the data material can improve the ability to sharpen focus and sharpen the purpose through the process. Today, all authors are registered nurse midwives with extensive knowledge and experience within postnatal care, the two last authors (ESV, ABVN) are experienced researchers. The Consolidated Criteria for Reporting Qualitative Research (COREQ) checklist (Tong et al., 2007) was used to strengthen the reporting of the data.

## 5 FINDINGS

A total of 11 midwives from nine different municipalities and regions across Norway were enrolled in the study. The ages of the midwives ranged from 38 to 59 years old (mean age: 46) and the time in which they had spent in the profession ranged from 3 to 30 years (mean amount of time: 12). Two main results were identified through the analysis: (1) the midwives adapted quickly to changes in postpartum care during the pandemic and (2) midwives saw the experience as an opportunity to re‐evaluate their practices.

### 5.1 The midwives adapted quickly to changes in postpartum care during the pandemic

The midwives described an abrupt stop in providing postpartum home visits as Norway introduced strict national measures on 12 March 2020 in order to combat the spread of the COVID‐19 coronavirus. As the midwives adapted to the pandemic, it was a common aim to continue providing new families with high‐quality care. Some described how face‐to‐face follow‐ups were limited to short meetings, in which the women would not always have time to breastfeed. It was believed that both breastfeeding frequency and both parents' sense of security may have been affected during the pandemic. It was noted that it seemed to become a concern among the women, that pregnancy, birth and the postpartum period were characterised by uncertainties, especially in relation to lack of involvement of partners. Midwives explained that there had been an increase in postpartum depression during the pandemic, including among non‐birthing parents. One midwife described postpartum care as follows:

At the start of the pandemic, we were no longer allowed to go on home visits… It wasn't okay. The quality [of care] was completely different. (Interview 11)

The midwives had to be creative in order to solve new and unexpected challenges that emerged as the pandemic progressed. The beginning of the pandemic was said to be a particularly demanding period, mainly characterised by strict national guidelines. The midwives thus had to find new ways of communicating and telephone contact quickly became a major part of their everyday work. Previous benefits of using and reading body language were now almost non‐existent. The midwives instead concentrated on how they could benefit from other skills, such as how to best explain what the mother could do to trigger the baby's natural feeding reflexes. The midwives' verbal explanations became increasingly important, and as a result, they noted rapid improvements in their own teaching and guidance skills. The midwives used phone and video consultations as temporary substitutes for postpartum home visits, and they mentioned several creative solutions they had to come up with in situations where opportunities were limited. Examples of new solutions included weighing a newborn using an ovenproof dish on a kitchen scale, or offering online video consultations for breastfeeding guidance. One midwife, who was unable to do home visits due to restrictions in her area, described one particular episode early on in the pandemic:

The woman hadn't been able to weigh the baby, so I went to the address and put the baby scale outside the door before I went back to my car. She picked up the baby scale. I waved to the siblings standing by the window and she showed me the baby though the window as well. The woman texted me the baby's weight and sent me a picture of the baby's eyes. She had a question related to the eyes, and then I guided her through breastfeeding on Facetime afterwards [i.e. social media]. She just wanted to know if the baby had latched on correctly. (Interview 2)

Initially, they called the women by phone as a substitute for the home visit, and other options such as video consultations were eventually implemented. In collaborative meetings within the collegium, videos were also considered a suitable alternative, as several municipalities went on to film their own breastfeeding and postpartum videos and prepared electronic packages consisting of information about the postpartum period to disseminate to the women and their partners. Some midwives felt they were working in a more digitally up‐to‐date manner, while others said that it felt strange to work behind a screen and not meet the women physically. Nevertheless, they concluded that it worked well for a period, but was not an adequate long‐term solution. Some midwives were equipped with electronic aids, such as iPads and computers that had access to the workplace network and journal system. One of the midwives described the change in service as follows:

So, I guess we've taken an exceptional crash course in digitalisation. (Interview 4)

### 5.2 Midwives saw the experience as an opportunity to re‐evaluate their practices

The first 14 days of the lockdown were described as chaotic. There was a lot of uncertainty among the midwives. They explained how everyone had to make their own rules and that no one knew exactly how to proceed with the postpartum care follow‐ups. It was generally referred to as an extraordinary time that no one was prepared for, one that was particularly demanding and despairing for the midwives. The uncertainties relating to their everyday work left them frustrated and confused. Several reported a lack of dialogue with managers and poor information flow between the healthcare services, and midwives felt alone. The need for midwifery management in postpartum care at the healthcare centres became particularly visible during this period. Several of the midwives missed information they were meant to have, as they themselves had to request updated procedures on infection control and the COVID‐19 pandemic. A desire for a common documentation system and access to the specialist healthcare service's guidelines was expressed. The midwives missed being represented in the emergency response groups too, as it meant they lacked a gateway to dialogue with the head doctor of the municipality and the specialist on infectious diseases. One midwife expressed her feelings about this as follows:

It was very… frustrating to be a midwife during this period. And, in a way… we had no direct meetings with the head doctor in the municipality, because it was somehow no one who took responsibility for the midwifery care services, except the things we initiated and carried out ourselves. (Interview 6)

Several of the midwives mentioned the fact that they were not afraid of being infected by COVID‐19 themselves. Rather, the idea of risking being the one who infected a newborn, a mother or other family members, frightened the midwives. Words such as fear, anxiety, stress, being terrified or frightened were used to describe such feelings of putting new families at risk. When possible, the midwives were careful to break potential chains of infection, such as when they kept physical distance with new families. The midwives explained how the fear of infecting families was of great concern to them, and therefore became an important reason for them to limit what they did in their private lives. To provide safe postpartum care, the midwives made changes to their own lives, by staying at home when possible and abstaining from using public transport. One of the midwives illustrated the fear of infecting others in the following way:

… it is one thing to be infected yourself. I think I can live with that, as long as I do not die from it. But to be the one who infects someone else… I do not think that is pleasant thought at all. (Interview 4)

The midwives used words such as ‘*demanding’* and ‘*challenging*’ when they talked about what it was like to work with postpartum care during the pandemic. They described how the risk of infection created an increased distance between them and the women. The midwives explained how their follow‐ups would have normally allowed for closeness between the midwife and the mother, both physically and mentally. The natural end to these follow‐ups often took place during home visits after childbirth. During the pandemic, it became difficult to ensure the same level of contact with the family and get a true idea of their well‐being. The midwives described physical contact as a key component of midwifery, and found it difficult to wear a face mask, as mimicry and body language are so important when communicating with women in these situations. Several midwives used the word ‘*hopeless’* when talking about guiding women through breastfeeding when they had to maintain a distance of two meters. This is how one of the midwives described one of their first home visits after the start of lockdown:

They were really happy when I came. However, both of us felt as if we almost did something illegal. So, keeping distance was important… You weren't as close as you really wanted… you should hardly touch anything. You sit on the edge of the chair… you don't touch the table. (Interview 11)

When the midwives looked back and reflected on the first year of the COVID‐19 pandemic, most of them were left with a feeling that the women and their families had been given quality care despite the many restrictions. The midwives felt that they might have done more than anyone could have expected them to do, referring to it as a challenging period. Several of the midwives mentioned that they had a particularly heavy workload during the pandemic period. They learned new ways of working, although this required what they called ‘*self‐drive*’. Several described that they had to adapt as they could not provide the usual hands‐on approach, meaning that they discovered a unique opportunity to observe the interaction between parents and newborns. According to the midwives, this practice empowered the parents and was therefore something the midwives wanted to continue with after the pandemic. Several concluded that the postpartum care had not suffered to too great an extent, mainly due to adjustments that they made. After the initial chaotic period, national restrictions became clearer, and one midwife noted the following:

‘Now, I have the experience, right… So, now I'm more confident in what I'm doing when it comes to prioritisation. And, as time goes by, we get better. I feel stronger now than I was before the pandemic, because this period has been something out of the ordinary. I think I have learned a lot, and I am ready for a new wave of COVID‐19. (Interview 9)

## 6 DISCUSSION

**FIGURE 1:**
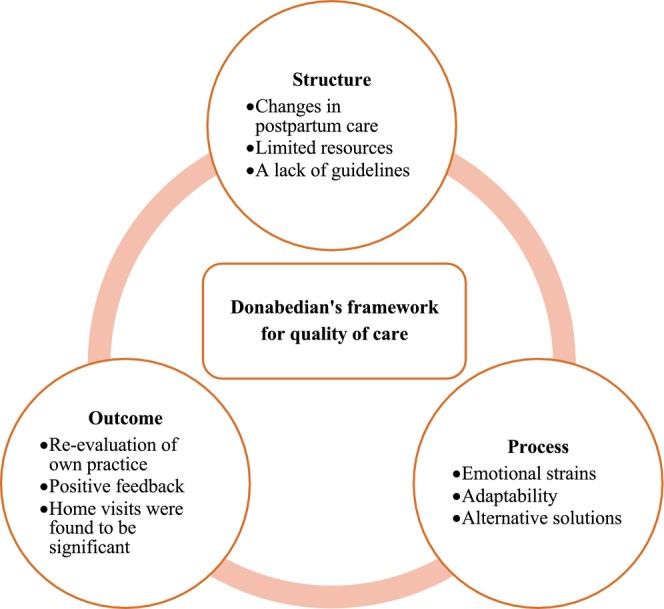
Illustrates the findings of the study relating to the components of Donabedian's framework.

In the current study, midwives described an abrupt stop in postpartum home visits during the first wave of the COVID‐19 pandemic; however, the midwives quickly found alternative solutions. The midwives were left to make important decisions, and while fearful of causing infection, they also felt the experience was an opportunity to re‐evaluate their own practices. To ensure the well‐being of both the mother and the baby, it is important to address any safety issues that are inherent to the way postpartum care is organised and carried out. Therefore, we discuss the midwives' experiences specifically in a high‐income setting, in light of Donabedian's framework for quality of care. The framework was chosen after analysing the study data, and it consists of the following three components: structure, process and outcome (Donabedian, 2005). Structure relates to the prerequisites required to achieve quality in healthcare services, such as its resources. Process relates to different aspects of the actions taken to enable quality services, for example building relations between caregiver and care recipient. Finally, outcome relates to the effect of said care on the recipient. Figure 1 illustrates the findings of the study relating to the components of Donabedian's framework.

### 6.1 Structure

Through interviews with midwives working with home‐based postpartum care in Norway, we found that they essentially had to adapt overnight and make major structural changes to the service they offered, at the time when the COVID‐19 pandemic struck the country. Furthermore, the midwives explained that they received few guidelines from management and that it was challenging to be left to make their own decisions regarding the planning and organisation of postpartum care. They reported missing a closer dialogue with the management, specifically the head municipal doctor and the infection control doctor, and to be included in the emergency response groups. The midwives in our study experienced the Norwegian postpartum care during the pandemic as rather disorganised, and they missed the collaboration they used to have across the healthcare centres. Similar to the findings in a pre‐pandemic Norwegian study (Levorstad et al., 2022), the midwives said that in many ways they felt ignored and deprioritised within their municipalities when changes were planned and implemented. A study on midwives' experiences on quality of care in the Netherlands during the COVID‐19 pandemic (Hijdra et al., 2022) reported that a task force including chairwomen from regional midwifery collaborations was established by the Royal Dutch Organisation for Midwives to organise midwifery care on regional levels. Results from the Dutch study showed that information from the taskforces were perceived as rapidly changing and unclear, which caused stress, confusion and frustration. A similar result is reported in a study from Northern Italy (Fumagalli et al., 2022) where the term “infodemic” was used to describe the overwhelming amount of rapidly changing information experienced by midwifes. These studies indicate the need for a balanced approach in decision making and information flow.

A qualitative study from the United States including 21 interviews with nurses about their experiences of caring for hospitalised COVID‐19‐positive patients in a hospital (Schroeder et al., 2020). In this study, the nurses described large and frequent changes in the structure of the service. However, the nurses described having a good dialogue with managers and infectious disease specialists during the pandemic. The nurses also had access to the relevant guidelines and procedures at the hospital (Schroeder et al., 2020). These results coincide with a case report evaluating the COVID‐19 response at a hospital in New York State, USA (Binder et al., 2021). The case report described how the management of a hospital made major necessary structural changes in the face of the pandemic, using the Donabedian framework to help implement them. The hospital management maintained acceptable dialogue with the employees. In response to frustration with constant changes, the management set up weekly digital meetings, in which employees could discuss the changes they were facing. Further, the employees had access to competence and learning portals where it was possible to find and receive professional updates as the changes were introduced. Looking at Donabedian's structural component, it appears that the midwives in our study had other and perhaps inferior resources and prerequisites for providing quality care, compared to the health workers in the two American studies (Binder et al., 2021; Schroeder et al., 2020). None of these studies examined the experiences of midwives delivering out‐of‐hospital post‐partum care, but nurses and health workers facing comparable situations and challenges.

Although the impact of the COVID‐19 pandemic on healthcare services and daily life is now declining, new epidemics or pandemics may still emerge in the future, thus suggesting a need for professional guidelines that could still ensure quality in the postpartum care. In a recent study from Australia, the researchers used questionnaires and interviews to investigate midwives' experiences of maternity care during the COVID‐19 pandemic, with the findings of this study also supporting a need for customised guidelines (Bradfield, Wynter, et al., 2021). Moreover, the knowledge acquired during this pandemic should be utilised to draw up such guidelines.

### 6.2 Process

In this study, the midwives described the transformation of their roles during this time as a challenging process in which they felt a great responsibility for the postpartum care they provided. The midwives noted that the initial period was particularly challenging, as new procedures would be implemented quickly and further work would be established with a high degree of uncertainty. Similar findings are described in a qualitative study conducted in Iran, where they examined healthcare professionals' experiences of working during the COVID‐19 pandemic, with most of participants reporting that they felt helpless, describing the situation as out of control (Ardebili et al., 2021). The process that the midwives in our study went through shows that they did make the necessary changes to meet the challenges introduced as a result of the pandemic. Similar to healthcare personnel from the qualitative study conducted in the United States (Schroeder et al., 2020), the midwives in the current study were able to quickly evaluate the situation and find alternative solutions. As stated in our results, digital follow‐ups became one of the first solutions and a temporary replacement for home visits in the earliest stage of the pandemic. According to a Norwegian white paper on quality of care and patient safety (Meld. St. 11, 2020–2021, p. 6), the healthcare services experienced a major digital boost in this time, and a clear goal to further utilise digital aids to provide home‐based services is outlined. The midwives in our study have illustrated exactly how such technology has been employed as a useful tool throughout the pandemic. It gave them flexibility in their everyday work, and they also commented on how digital postpartum follow‐ups could have a financial advantage and save them time in regard to travel. However, as discussed by van den Berg et al. (2022) rapid innovations which more or less appeared overnight should be carefully evaluated post‐crisis.

COVID‐19 pandemic also led the midwives to go through a process of great emotional stress. This was especially prevalent in regard to the fear of infecting new families, which significantly impacted their everyday lives. Similar findings have been presented in other studies that examined health professionals' experiences with the pandemic (Ardebili et al., 2021; Bradfield, Wynter, et al., 2021; Hijdra et al., 2022). These studies revealed the fear held by the healthcare professionals in being a carrier of the virus and thus infecting patients, colleagues and their own families (Ardebili et al., 2021; Bradfield, Wynter, et al., 2021). It is conceivable then that a lack of information and knowledge about the virus may have contributed to the increased fear of infecting others. A qualitative study conducted in Spain examined midwives' experiences of providing maternity care for women infected with the COVID‐19 virus (González‐Timoneda et al., 2021). The midwives had a lack of experience in how to prepare and remove infection control equipment and clothing and felt a great deal of uncertainty associated with this. Furthermore, they described that an increased focus on infection control came at the expense of the amount and quality of care intended for mothers and babies. This was also experienced by the midwives in our study; they explained that mimicry and body language are an important part of how they communicate with the woman, which was hindered by the infection control equipment they had to use. The midwives were therefore challenged to find alternative ways of communicating and described what they had to do to achieve this as that of being a creative process. The midwives wanted to make the best of the situation and preserve the positive engagement they previously had with their patients. This corresponds to the results of the study conducted in Australia (Bradfield, Wynter, et al., 2021), in that the results of our study underline the importance that midwives, like other healthcare professionals, have adapted to the pandemic and significantly extended what they do in their roles in order to provide quality care.

### 6.3 Outcome

By taking part in this study, the midwives were given the opportunity to look back on the first wave of the COVID‐19 pandemic and re‐evaluate their own practices. They felt that the outcome of the changes made to the structure and process of their work was mainly positive. As they adjusted the postpartum care they could provide, and made the necessary changes, they evaluated that the care given was still of high quality. As this study examines midwives' experiences with providing home‐based postpartum care during the COVID‐19 pandemic, it will be difficult to say anything about the health effects the pandemic has had on the postpartum women. However, the midwives said that they received positive feedback from the women, and they felt that the families were satisfied with the postpartum care they received at that time. In the current study, the midwives emphasised that they had seen how important the midwives' home visits were to the women. A study including 21,027 women (Lazzerini et al., 2022) revealed that women who underwent labour in Europe during the first year of the COVID‐19 pandemic experienced not being allowed a companion of choice while in hospital (31.1%), insufficient number of health workers (31.8%) and receiving inadequate breastfeeding support (19.4%). In a qualitative study conducted in Norway before the pandemic, nine women were interviewed about their experiences of their midwife's home visits following an early discharge from hospital (Aaserud et al., 2017). In the study, the midwives' home visits were described as meaningful and informal, and created peace and security during a hectic postpartum period, and it could provide the opportunity to meet individual needs and strengthen the woman's experience of mastery (Aaserud et al., 2017). These findings show a need to emphasis on follow up after discharge from hospital.

The midwives in our study said that they experienced a difference in the quality with providing home‐based postpartum care and postpartum care provided over the phone and virtually, and at the health centres. Important observations, such as attachment and the interaction between the parents and the baby, were missed. It was therefore important for the midwives to meet the women face‐to‐face in their homes. In a qualitative study conducted in Sweden, they examined 14 women's perceptions of safety in the first period following on from childbirth (Persson et al., 2011). The women in the study highlighted the importance of their partner's presence during the home visit. When the partner was accepted and included in the care of the newborn child, it promoted a positive and equal cooperation in their new family dynamic (Persson et al., 2011). The midwives in our study found that the woman's partner was often side‐lined during the pandemic, and they expressed a desire to provide quality care to the whole family. Similar findings were presented in a large Norwegian study exploring 804 women's experiences of giving birth and becoming parent during the COVID‐19 pandemic (Eri et al., 2022).

All of the midwives included in the current study described their experiences of the COVID‐19 pandemic as enlightening, even though they have rather not had to experience it in the first place. However, the midwives explained that, through the pandemic, they had evolved, making them better prepared to handle a similar situation in the future. This is also evident in the study from Australia, which emphasises the importance of using the midwives' experiences so as to be ready when facing another potential pandemic in the future (Bradfield, Wynter, et al., 2021).

### 6.4 Implications and recommendations for practice

Clinicians should be included when planning postpartum care services, and the lessons learned from the COVID‐19 pandemic should not be forgotten. Considering changes in current practise, with an increasing number of new families being discharged early after birth (Jones et al., 2021), the WHO recommend that educational materials, such as written and/or digital information and illustrations for semi‐literate populations are made available for families when discharged (WHO, 2022). In line with updated recommendations from the WHO, situations where home‐based care is not feasible or preferred, outpatient postpartum care contacts are recommended (WHO, 2022).

### 6.5 Limitations of the study

Due to travel restrictions during the COVID‐19 pandemic, it was not possible to conduct face‐to‐face interviews. When conducting interviews digitally, it may be the case that important elements, such as the interviewees' mood and nonverbal expressions are not truly captured. Nevertheless, interviews conducted digitally can also be considered a strength as it allowed us to include a more heterogeneous sample, in that we were able to include midwives from a range of municipalities of different sizes and demographics across Norway. The current study was limited to the experiences of midwives who were members of a specific closed Facebook group for midwives in Norway. Self‐recruitment may have affected our findings; therefore, interpretation of results and transferability must be made with caution. One study from 2022 (Mi et al., 2022) highlights ethnic disparities in postpartum care access after the onset of the pandemic. While highly relevant, such disparities were not explored in the current study.

### 6.6 Recommendations for further research

In a time of change, where an increasing number of families receive home‐based postpartum care in high‐income countries, researchers need to plan and conduct studies with different perspectives and designs with the overarching aim of promoting health and well‐being for all mothers, babies and their families. Such research would be in line with recommendations from the WHO (2022).

## 7 CONCLUSION

In conclusion, this study illuminates the resilience and adaptability demonstrated by midwives in navigating the exceptional challenges brought about by the first wave of COVID‐19 pandemic. The findings emphasise the essential role of face‐to‐face interactions in postpartum care, recognising the inherent value of physical presence and non‐verbal communication. Simultaneously, the study acknowledges the utility of technological interventions as viable alternatives in circumstances where direct access is limited. By shedding light on the experiences of midwives during this unprecedented period, this research makes a valuable contribution to the continuous pursuit of enhancing and optimising postpartum care delivery, particularly in the context of unforeseen circumstances and disruptions. This study highlights the importance of integrating multiple disciplines when planning postpartum care services. The valuable insights gained from lessons learned may have a lasting influence on the postpartum care system, empowering it to tackle unforeseen challenges both today and in the future.

## AUTHOR CONTRIBUTIONS

The first authorship was shared between Hanne Marie Akselsen and Emilie Hanssen Leknes, and the last authorship was shared between Eline Skirnisdottir Vik and Anne Britt Vika Nilsen. Hanne Marie Akselsen and Emilie Hanssen Leknes conducted and transcribed the interviews as a part of their master's degrees in Midwifery. Tone Engen, Eline Skirnisdottir Vik and Anne Britt Vika Nilsen teach and supervise the Midwifery education program at Western Norway University of Applied Sciences. Hanne Marie Akselsen, Emilie Hanssen Leknes, Eline Skirnisdottir Vik and Anne Britt Vika Nilsen made substantial contributions to conception and design, or acquisition of data, or analysis and interpretation of data. Hanne Marie Akselsen, Emilie Hanssen Leknes, Tone Engen, Eline Skirnisdottir Vik and Anne Britt Vika Nilsen were involved in drafting the manuscript or revising it critically for important intellectual content. All authors have participated sufficiently to take public responsibility for appropriate portions of the content, and further; to be accountable for all aspects of the work in ensuring that questions related to the accuracy or integrity of any part of the work are appropriately investigated and resolved. All authors have given final approval of this final version to be published.

## FUNDING INFORMATION

No funding.

## CONFLICT OF INTEREST STATEMENT

The authors declare that they have no competing interests.

## Supporting information


Data S1.

## Data Availability

Data available on request and if new approval from NSD and REK.
