# Supplementary material for: Midwives' experiences with providing home‐based postpartum care during the COVID‐19 pandemic: A qualitative study
Source: Nurs Open. 2023 Aug 31;10(11):7333–42. doi: 10.1002/nop2.1986 (PMC10563426; doi:10.1002/nop2.1986)
Supplement: Supplementary file 1 — Data S1. [file NOP2-10-7333-s001.docx]

**Interview guide**

**Introduction:**

Prior to the interview we do introductions and acknowledge the informants for participating before proceeding to present the aim of the interview.

The aim of the study was to explore midwives' experiences with home-based postpartum care during the COVID-19 pandemic in Norway.

**Information on**

How long the interview will last

Remind of statement of consent

Information on possibility to take breaks end/or stop the interview

Do technical sound and vocal tests

**Information on the informant midwife**

What is your age

What is your percentage of employment?

How many years have you practiced as a midwife?

How many years have you been employed in community health care services?

In which municipality are you currently employed?

Where were you employed previously?

How many women do you have a responsibility for in your municipality?

How many births is connected to the community health care service where you work?

Have you executed home visits both before and after the changes due to COVID-19?

**Main questions**

1. Please, tell us about how you experienced going on home visits, prior to the COVID-19-pandemic?
2. Please tell me about the purpose of a home visit. How do you and your colleagues work to meet this purpose?
3. Tell me about the changes that has been done at the community health care centre where you work due to COVID-19.
4. Can you tell me something positive about these changes? – Why was it positive?
5. Can you tell me something negative about these changes? – Why was it negative?
6. Tell me about one of your home visits during COVID-19.
7. Please tell me about the first home visit you were on after the pandemic struck, and how do you experience home visits now? Thinking back to March/April?
8. How do you experience working as a midwife during the pandemic on a personal level?

Summarizing during the interview: maybe get more elaborate answers to the questions

1. How do you cooperate with the community nurse/Hospital/ General Practitioner (GP).
2. Tell me about what kind of reactions families with a newborn has given on the service provided to them during the COVID-19 pandemic. Feel free to give examples.

**Finishing questions**

- Is there something you wish to elaborate or talk about that we have not covered yet?

**Follow-up questions:**

- Please tell me more about ...
- Please explain/elaborate …
- What/how did you feel/what did you think of …
